# Independently evolved and gene flow‐accelerated pesticide resistance in two‐spotted spider mites

**DOI:** 10.1002/ece3.4916

**Published:** 2019-02-03

**Authors:** Pan Shi, Li‐Jun Cao, Ya‐Jun Gong, Ling Ma, Wei Song, Jin‐Cui Chen, Ary A. Hoffmann, Shu‐Jun Wei

**Affiliations:** ^1^ Institute of Plant and Environmental Protection Beijing Academy of Agriculture and Forestry Sciences Beijing China; ^2^ School of BioSciences, Bio21 Institute The University of Melbourne Parkville Victoria Australia

**Keywords:** bifenazate, evolution, genetic structure, resistance, *Tetranychus urticae*

## Abstract

Pest species are often able to develop resistance to pesticides used to control them, depending on how rapidly resistance can emerge within a population or spread from another resistant population. We examined the evolution of bifenazate resistance in China in the two‐spotted spider mite (TSSM) *Tetranychus uticae *Koch (Acari: Tetranychidae), one of the most resistant arthropods, by using bioassays, detection of mutations in the target *cytb* gene, and population genetic structure analysis using microsatellite markers. Bioassays showed variable levels of resistance to bifenazate. The *cytb* mutation G126S, which confers medium resistance in TSSM to bifenazate, had previously been detected prior to the application of bifenazate and was now widespread, suggesting likely resistance evolution from standing genetic variation. G126S was detected in geographically distant populations across different genetic clusters, pointing to the independent origin of this mutation in different TSSM populations. A novel A269V mutation linked to a low‐level resistance was detected in two southern populations. Widespread resistance associated with a high frequency of the G126S allele was found in four populations from the Beijing area which were not genetically differentiated. In this case, a high level of gene flows likely accelerated the development of resistance within this local region, as well as into an outlying region distant from Beijing. These findings, therefore, suggest patterns consistent with both local evolution of pesticide resistance as well as an impact of migration, helping to inform resistance management strategies in TSSM.

## 1 INTRODUCTION

Human activities can impose very strong selection on natural populations of animals and plants (Hendry et al., 2011; Hoffmann & Parsons, 1997). One of the most obvious selective pressure comes from the application of pesticides in agriculture ecosystems. Target species are often able to evolve resistance soon after a new compound is introduced (Denholm & Rowland, 1992; Hawkins, Bass, Dixon, & Neve, 2018; Roush & McKenzie, 1987; Van Leeuwen, Tirry, Yamamoto, Nauen, & Dermauw, 2015; Van Leeuwen, Vontas, Tsagkarakou, Dermauw, & Tirry, 2010). More than 580 arthropod species have evolved resistance to at least one pesticide (Sparks & Nauen, 2015). A fundamental question in the evolution of pesticide resistance is the origin of resistance mutations in natural populations (Ffrench‐Constant, 2007), whether they emerge within a population from standing genetic variation or as new mutations, versus being introduced into it through migration. Answers to this question can contribute to insecticide resistance management (IRM) programs and theories of adaptive evolution more generally (Daborn & Le Goff, 2004; Hawkins et al., 2018; MacLean, Hall, Perron, & Buckling, 2010; Neve, Busi, Renton, & Vila‐Aiub, 2014).

Resistance alleles can be maintained in standing genetic variation in populations before selection for resistance (Barrett & Schluter, 2008) or emerge from new mutations subsequent to a selective challenge (Woods, Schneider, Winkworth, Riley, & Lenski, 2006). For contemporary evolution occurring on the time frame of less than a few hundred years (Hendry & Kinnison, 1999), standing genetic variation generally contributes more adaptive mutations than new mutations (Hendry et al., 2011). In pest insects, resistance mutations nevertheless can arise from new mutations as evidenced by cases of target‐site resistance (Riveron et al., 2014; Weetman et al., 2015), as well as from standing genetic variation (Troczka et al., 2012) or a combination of processes (Hartley et al., 2006; Rose et al., 2011) as summarized by Hawkins et al. (2018).

Multiple origins of resistance mutations are evident from resistance being conferred by the same site mutation in different species/subspecies (Anthony, Brown, Markham, & Ffrenchconstant, 1995; Thompson, Steichen, & Ffrench‐Constant, 1993; Weill et al., 2003), and when resistance to the same pesticide involves different mutations in different populations (Andreev, Kreitman, Phillips, & Beeman, 1999). However resistance allele originating from single sites can also be dispersed globally (Daborn et al., 2002; Raymond, Callaghan, Fort, & Pasteur, 1991), particularly in highly mobile pest species in agricultural ecosystems (Cao et al., 2017). The widespread dispersal of resistance alleles can increase the risk of resistance developing in distant populations (Osakabe, Uesugi, & Goka, 2009).

Although the development of resistance to pesticides is well established, less is known about the origin and dispersal of resistance mutations (Hawkins et al., 2018). The genetic basis of resistance needs to be understood to track the origin of resistance mutations (Daborn & Le Goff, 2004; Hawkins et al., 2018). For this reason, most work on the origin of pesticide resistance has been based on target‐site resistance (Daborn & Le Goff, 2004; Raymond et al., 1991; Troczka et al., 2012; Weetman et al., 2015).

In this study, we used the two‐spotted spider mite (TSSM) *Tetranychus uticae *Koch (Acari: Tetranychidae), one of the arthropods with very high levels of resistance (Ilias, Vassiliou, Vontas, & Tsagkarakou, 2016), as a model species to investigate the origin and dispersal of bifenazate resistance mutations. Bifenazate (Uniroyal Chemical Company, Inc., USA) is a new type of acaricide for controlling spider mites (James, 2002; Van Leeuwen, Tirry, & Nauen, 2006). It works as a cytochrome b (*cytb*) Qo‐pocket inhibitor, targeting the mitochondrial cytochrome bc1 complex (complex III; Van Leeuwen et al., 2008). This mode of action was recently supported by a study revealing maternally inherited cross‐resistance between bifenazate and acequinocyl, an HONQ acaricide (Van Nieuwenhuyse, Leeuwen, Khajehali, Vanholme, & Tirry, 2009). Previous studies showed the resistance of TSSM to bifenazate is mainly related to *cytb* nonsynonymous mutations including amino acid substitutions of G126S, I136T, and S141F (TSSM numbering) located in the cd1 helix or the P262T mutation near the ef helix aligning the cytochrome bc1 enzyme pocket (Van Leeuwen et al., 2008). A combination of two mutations (G126S and I136T, G126S and S141F) in cd1 helix or the P262T mutation seems to be necessary to confer extremely high resistance in TSSM in the laboratory. The G126S mutation alone only confers a lower level of resistance. However, this mutation may be coupled with a second mutation (I136T or S141F) to cause extremely high resistance.

Bifenazate was released in China in 2013 (Gong et al., 2013). It is a reliable pesticide for the control of TSSM (Xu et al., 2018), which is a serious pest on many crops, especially strawberry. After five years of usage, bifenazate has become less effective in several regions, suggesting that resistance development was in progress. Prior to the usage of bifenazate in the Beijing area, a resistance‐related mutation G126S in one individual from 288 individuals was detected in natural populations (Gong et al., 2014), suggesting standing genetic variation for bifenazate resistance in TSSM in this area. Parallel evolution of bifenazate resistance mediated by mutation of cytochrome b was found the citrus red mite, *Panonychus citri* (Van Leeuwen et al., 2011). The extensive transport of strawberry seedlings across China possibly enables high levels of gene flow among TSSM populations with the potential to spread the resistance mutation.

In this study, we examined the resistance status of TSSMs to bifenazate and detected mutations in the *cytb* gene in field populations across China. The population genetic structure of TSSMs was investigated based on microsatellites to trace the evolution and dispersal of resistance mutations among populations. We assumed that de novo mutations of TSSMs arising independently in China, based on the presence of resistance mutation prior to the usage of bifenazate (Gong et al., 2014), and parallel evolution of resistance mutation to bifenazate in relative spider mite species (Van Leeuwen et al., 2011), and high genetic structure among populations of TSSM (Chen, Zhang, Du, Jin, & Hong, 2016; Navajas et al., 2002; Sun, Lian, Navajas, & Hong, 2012). The pattern of resistance evolution revealed in our study can help facilitate effective IRM and provides information on processes involved in resistance evolution against this pesticide.

## 2 MATERIALS AND METHODS

### 2.1 Sample collection and rearing

In total, ten populations of spider mites were collected from strawberry fields across seven provinces of China from February to May in 2017 (Table 1). When collecting the spider mites, we respectively chose about thirty scattered points from every field to avoid the collection of close relatives. Some of the spider mites collected were preserved in absolute ethanol for molecular analysis; the remaining mites were transferred to bean plants (*Phaseolus vulgaris* L.) to be cultivated for bioassays in the laboratory. TSSM does not readily move from strawberry leaves onto bean leaves, but once on bean leaves the mites are easily moved for bioassays as described below. A susceptible strain of TSSM maintained at the Institute of Vegetables and Flowers, Chinese Academy of Agricultural Sciences, was used as a control in the bioassays. This population had been reared in the laboratory for ten years without contacting any pesticides. Populations of TSSM were reared at 25 ± 0.5°C with 60% relative humidity and a 16:8 (light:dark) photoperiod.

**Table 1:** Information on the 10 field populations of *Tetranychus urticae* used in the study

Code	Collection location	Longitude (°E)	Latitude (°N)	*N*, *cytb*	*N*, microsatellite
SCCD	SiChuan Province, Chengdu	104.0679	30.6799	25	21
HNCS	Hunan Province, Changsha	112.9632	28.0395	13	9
AHHN	Anhui Province, Huainan	116.8612	32.8090	18	16
ZJJX	Zhejiang Province, Jiaxing	120.9405	30.8844	17	15
SXYQ	Shanxi Province, Yangquan	113.3605	38.1727	23	14
SDRZ	Shandong Province, Rizhao	118.9034	35.8347	21	15
BJC1	BeiJjing, Changping	116.4620	40.2134	27	19
BJC2	BeiJing, Changping	116.4256	40.2008	18	22
BJCW	BeiJing, Wandeyuan	116.2165	40.2217	23	23
BJDX	BeiJing, Daxing	116.4252	39.6528	22	26

Note

*N*: number of individuals examined in each population for *cytb* gene sequencing and microsatellite genotyping.

### 2.2 Bioassays

The bioassay was carried out using a slide‐dip method with 43% bifenazate as described previously (Gong et al., 2013). In brief, TSSM adults were stuck onto one end of a slide with double‐sided sticky tape. After 2 hr, inactive and dead individuals were removed with an insect needle, and only the active adult mites remained, leaving 20–30 individuals per slide. Based on preliminary tests, seven concentrations of 43% bifenazate from 15.625 to 1,000 mg/L were diluted using water (containing 0.1% Triton X‐100). Water containing 0.1% Triton X‐100 was used as a control. Four replications were conducted for each treatment. After dipping in different treatments for 5 s, the slides with TSSM were dried naturally at room temperature and kept in the Animal Breeding System (LP‐80CCFL‐6AR/6ARS, NK system) at 25 ± 0.5°C, 60% relative humidity and a 16:8 (light:dark) photoperiod. Mortality was scored after 48 hr.

### 2.3 Molecular analysis

Ten field populations of TSSM were used for sequencing of mitochondrial genes (both male and female) and genotyping of microsatellite loci (female). A rapid method was used to extract DNA from individual specimens (Mardulyn et al., 2013). Individuals stored in absolute ethanol were air‐dried and picked into 0.2‐ml tube containing 20 µl of lysis buffer (10 mM of Tris–HCl pH 8.2, 50 mM of KCl, 2.5 mM of MgCl_2_, 0.45% Tween‐20, 0.01% gelatin, 60 μg/ml proteinase K) and homogenized using 20‐μl pipette tips. After centrifugation, each tube of liquid was then incubated at −80°C for 30 min, at 65°C for 1 hr and finally at 95°C for 15 min. The DNA templates were stored at −20°C prior to usage.

Cytochrome c oxidase subunit I (*cox1*) was amplified for species identification of the spider mites collected from the field (Xie, Hong, & Xue, 2006). Based on the complete mitochondrial genome of TSSM (GenBank accession no. EU345430; Van Leeuwen et al., 2008), species‐specific primers of LepF‐TU (5′‐ATTCAACCAATCATAAAGATATTGG‐3′) and LepR‐TU (5′‐TAAACTTCTGGATGTCCAAAAAATCA‐3′) were modified from LepF and LepR (Hebert, Penton, Burns, Janzen, & Hallwachs, 2004) using Primer3 web version 4.1.0 (http://primer3.ut.ee/). For each population, eight individuals were randomly selected for molecular identification.

The complete sequence of *cytb* gene (2,846 bp), which was found as the target gene for bifenazate resistance in TSSM, was amplified and sequenced from 207 adults (Table 1) using primer pairs cytbF (5′‐ATACCGAAACCGTGGAAA‐3′) and cytbR (5′‐TCTTGCTTTTAGTCGYTGAT‐3′) designed based on the complete mitochondrial genome sequence of TSSM (Van Leeuwen et al., 2008). The polymerase chain reaction (PCR) volume of *cox1* gene was set to 15 µl containing 7.5 µl of Master Mix (Promega, Madison, WI, USA), 0.3 µl of each primer (10 mM), 2 µl of DNA template, and 4.9 µl of ddH_2_O. The PCR volume of *cytb* gene was set to 15 µl containing 1.5 µl of 10 × buffer (Mg^2+^ Plus), 1.5 µl of dNTP, 0.6 µl of each primer (10 mM), 0.2 µl of LA Taq enzyme (TaKaRa), and 2 µl of DNA template and ddH_2_O added. The PCR program was conducted using the Mastercycler pro system (Eppendorf, Germany) under the following conditions: one cycle of predenaturation at 96°C for 2.5 min; 35 cycles of denaturation at 96°C for 30 s, annealing at 44°C (*cox1*) or 50.6°C (*cytb*) for 30 s, and extension at 60°C for 1 min (*cox1*) or 3 min (*cytb*), followed by a final extension at 60°C for 10 min. The amplified PCR products were sequenced using a primer walking strategy on an ABI 3730xl sequencer (Applied Biosystems) with the primer of LepF‐TU for *cox1* and bidirectional primers of cytbdiaR1 (5′‐GAAACAAAAATTATTATTCCCCCAAC‐3′) and cytbdiaR2 (5′‐GGTACARATCGTAAAATTGC‐3′) for *cytb*.

To reveal the population genetic structure of TSSM, we chose five microsatellite loci which had proved to be stably amplified and with a high level of polymorphism (Ge, Sun, Cui, & Hong, 2013). We added a PC‐tail (Primer tail C; 5′ CAGGACCAGGCTACCGTG 3′) to the 5′ end of the candidate forward primers to improve amplification efficiency and reduce cost. A fluorescence‐labeled PC‐tail was added to the PCR volume to form a three‐primer amplification system (Blacket, Robin, Good, Lee, & Miller, 2012; Schuelke, 2000). In total, 180 female adults were genotyped from 10 populations (Table 1).

The final amplification volume was 10 µl, including 0.5 µl of template, 5 µl of Master Mix (Promega), 0.08 µl of PC‐tail‐modified forward primer (10 mM), 0.16 µl of reverse primer (10 mM), 0.32 µl of fluorescence‐labeled PC‐tail (10 mM), and 3.94 µl of ddH_2_O. The amplification program was set under the following conditions: 2 min at 94°C; 35 cycles of 30 s at 94°C, 45 s at 52°C, and 45 s at 72°C, followed by a final 10‐min extension at 72°C. The amplified PCR fragments were analyzed on an ABI 3730xl DNA Analyzer (Applied Biosystems) using the GeneScan 500 LIZ size standard (Applied Biosystems). Genotyping data were identified, and errors were corrected by GeneMapper version 4.0 Micro‐Checker (Oosterhout, Hutchinson, Wills, & Shipley, 2004).

### 2.4 Bioassay data analysis

The dose–response curve (DRC) model parameters, the lethal concentration 50 (LC_50_), the lethal concentration 95 (LC_95_) values, and their 95% confidential limits were calculated by probit regression using the R package *drc* v3.0‐1 (https://www.rdocumentation.org/).

### 2.5 Species identification, mutation, and population genetic diversity analysis

For mitochondrial genes, we checked and revised sequencing using CHROMAS Pro v2.1.3 software (Ibeagha‐Awemu, Akwanji, Beaudoin, & Xin, 2014). Sequencing results of *cytb* from both directions were assembled using Lasergene 7's SeqMan software (DNASTAR, Madison, WI, USA). Gene sequences were aligned with Clustal W2 (Larkin et al., 2007) implemented in MEGA7 (Kumar, Stecher, & Tamura, 2016). Species were identified by BLAST using haplotypes of the *cox1* gene as a query in GenBank. We downloaded all available *cytb* haplotypes of TSSM from GenBank for comparison of site mutations (Table 2). Mutations of *cytb* genes were checked in DNASP 6.0 software (Librado & Rozas, 2009).

**Table 2:** All haplotypes of the mitochondrial *cytb* gene of *Tetranychus urticae* identified in this study and retrieved from GenBank

Haplotype	GenBank accession no.	Location/population of presence	Mutation (protein/gene)
Hap1	MH837177	AHHN, HNCS	A269V/C806T
Hap2	MH837178, KJ729022	AHHN, HNCS, SDRZ, SXYQ, ZJJX, SCCD	Wild type
Hap3	MH837179	BJ, SCCD	G126S/G376A*
Hap4	MH837180	ZJJX	I86M/T258A
Hap5	EU345430	Laboratory	Wild type (susceptible)
Hap6	EU556747, EU556750	Hoek van Holland, the Netherlands, Tuil, the Netherlands	G126S/G376A* and I136T/T407C*
Hap7	EU556748	Hoek van Holland, the Netherlands	G126S/G376A*
Hap8	EU556749, FJ196445	Nieuwveen, the Netherlands.Ghent, Belgium	P262T/C784A*
Hap9	EU556751	Laboratory	Wild type (susceptible)
Hap10	EU556752	Ghent, Belgium	Wild type
Hap11	EU556753	Brussels, Belgium	Wild type
Hap12	EU556754	Selected from LS‐VL	G126S/G376A* and S141F/C422T*
Hap13	FJ196444	Laboratory	Wild type

Note

The mutations marked with a star are the sites associated with resistance to bifenazate.

For microsatellite data, statistics of genetic diversity such as allele frequencies, allele numbers (*A*_T_), observed heterozygosity (*H*_o_), and expected heterozygosity (*H*_e_) were estimated by the macros Microsatellite Tools (Park, 2001). Tests of Hardy–Weinberg equilibrium (HWE) at each locus, estimation of *F*_ST_ between population pairs and *F*_IS_ for each population were performed with GENEPOP version 4.2.1 (Rousset, 2008). Allele richness (*A*_R_) was calculated by FSTAT V2.9.3 (Goudet, 2002).

### 2.6 Population genetic structure analysis

Population genetic structure was analyzed based on microsatellite loci. First, phylogenetic relationships among the populations were inferred with POPTREE2 (Takezaki, Nei, & Tamura, 2010) using the neighbor‐joining (NJ) method (Saitou, 1987). Second, population differentiation was identified using the Bayesian analysis of population genetic structure (BAPS) analysis implemented BAPS version 6.0 (Cheng, Connor, Sirén, Aanensen, & Corander, 2013). We performed ten repeat runs of various *K* values (from 1 to 10). Additionally, the discriminant analysis of principal components (DAPC) was performed using R package *adegenet* version 2.0.1 (Jombart, 2008). This method does not rely on any biological hypothesis and provides complementary results to BAPS.

### 2.7 Correlation analysis

Isolation by distance (IBD) analysis was performed to evaluate the correlation of pairwise genetic differentiation (*F*_ST_) and geographic distance in TSSM populations using a Mantel test implemented in the R package *ade4* (Elbrecht et al., 2014) with 10,000 permutations. A correlation between resistance differentiation and pairwise genetic differentiation (*F*_ST_) was computed as a Mantel test to investigate the relationship between bifenazate resistance and population differentiation. Additionally, the correlation between *Q*‐matrices calculated from STRUCTURE analysis and resistance ratio to bifenazate was analyzed.

### 2.8 Gene flow analysis

We used BAYESASS 3.0.4 (Wilson & Rannala, 2003) to calculate the migration rates between population pairs of TSSM. Preliminary runs (10,000,000 steps) were conducted to adjust mixing parameters for allele frequencies and inbreeding coefficients, after which ten longer runs of 100,000,000 steps with different start seeds were performed. The trace outputs of ten longer runs were combined using Tracer 1.6 (Rambaut, Drummond, Xie, Baele, & Suchard, 2018) to calculate mean migration with a burn‐in of 50,000,000.

## 3 RESULTS

### 3.1 Varied levels of resistance to bifenazate

**Figure 1:**
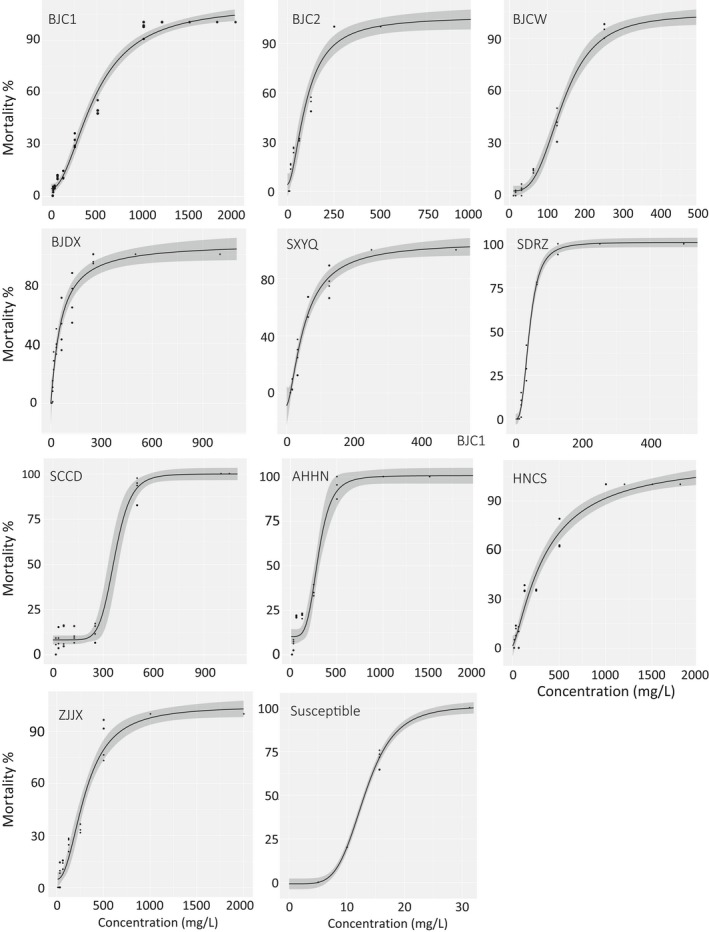
Dose–response cures of the 10 field and one susceptible *Tetranychus urticae* populations to bifenazate

Molecular identification based on the *cox1* gene indicated that all specimens randomly selected from the tested populations were TSSM. Compared with susceptible population whose LC_50_ is 13.12 mg/L, field populations of TSSM had variable levels of resistance to bifenazate (Table 3, Figure 1). Based on the resistance ratio, resistance status of the tested populations was classified into three levels. A medium level of resistance was present in populations from Beijing (BJC1, BJC2, BJCW, and BJDX) and Sichuan, with resistance ratios between 23.02 and 36.21. A low level of resistance was present in a population from Anhui and Hunan, with resistance ratios of 10.75 and 7.85, respectively. A decline level of susceptibility to bifenazate was found in populations from Zhejiang, Shanxi, and Shandong showed compared to susceptible population.

**Table 3:** Estimated lethal concentrations and resistance ratio for each population of *Tetranychus urticae *tested. For population codes, see Table 1

Population	LC_50_ (95% CI)	LC_95_ (95% CI)	RR
SCCD	475.03 (421.27–528.80)	2061.16 (1,350.43–2,771.90)	36.21
HNCS	103.02 (81.92–124.11)	534.81 (208.99–860.63)	7.85
AHHN	141.05 (130.99–151.12)	349.13 (252.65–445.60)	10.75
ZJJX	58.71 (36.56–80.86)	787.88 (35.83–1539.93)	4.47
SXYQ	53.50 (41.64–65.36)	367.92 (130.23–605.61)	4.08
SDRZ	41.10 (38.21–44.00)	116.25 (95.10–137.39)	3.13
BJC1	368.03 (333.47–402.59)	539.58 (476.85–602.31)	28.05
BJC2	302.06 (271.58–332.55)	590.10 (377.30–802.91)	23.02
BJCW	361.33 (266.25–456.40)	3,489.86 (291.49–6,688.24)	27.54
BJDX	307.41 (269.08–345.74)	1,154.97 (630.49–1679.45)	23.43
Susceptible	13.12 (12.70–13.53)	23.58 (21.75–25.40)	1.00

Note

Shading indicates a higher RR ratio. For population codes, see Table 1.

CI: confidence interval; LC_50_: lethal concentration that leads to 50% mortality; LC_95:_ lethal concentration that leads to 95% mortality; RR: resistance ratio calculated based on LC_50_.

### 3.2 Resistance mutations in the *cytb* gene

**Figure 2:**
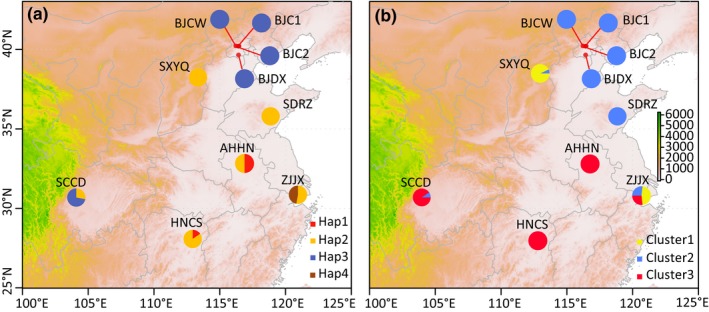
Collection map, distribution of four mitochondrial *cytb* gene haplotypes in 10 populations of *Tetranychus urticae* (a) and BAPS analysis of population genetic structure based on microsatellite loci (b). Population codes are listed in Table 1

**Figure 3:**
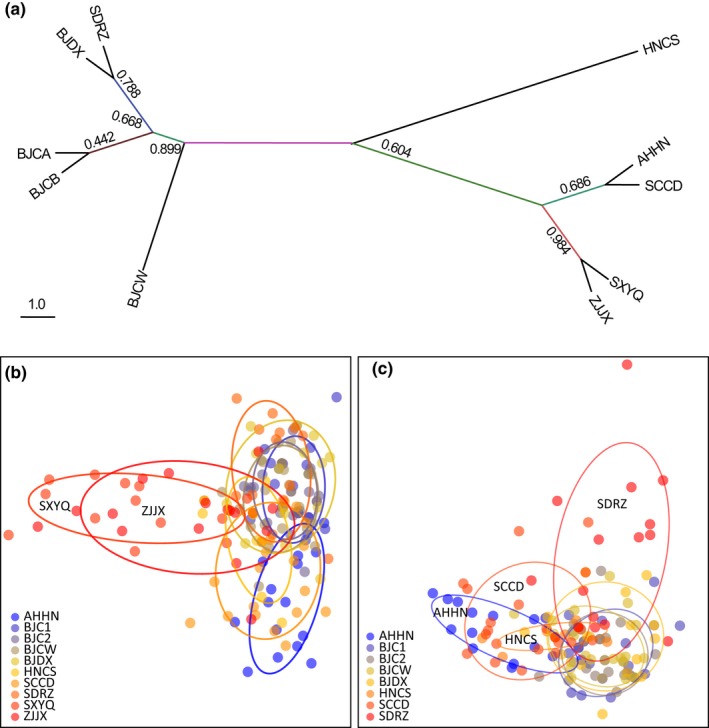
Mutations of the mitochondrial *cytb* gene in all available haplotypes of *Tetranychus urticae* identified in our study and downloaded from GenBank. The first four haplotypes marked with stars and three site mutations indicated by blue points are identified in this study; five sites marked by red triangles are previously identified mutations that are related to bifenazate resistance (Van Leeuwen et al., 2008); four sites marked by red points are conserved sites of *cytb* across multiple taxa (Van Leeuwen et al., 2008). The last nine haplotypes were retrieved from previously published references as shown in Table 2

In total, four haplotypes of *cytb* gene were identified from 10 populations of TSSM (Figures 2a and 3, GenBank accession numbers: MH837177–MH837180). Hap2 was previously reported (GenBank accession no. KJ729022) and widely distributed in low‐level resistance and susceptible declined populations of Anhui, Hunan, Zhejiang, Shandong, and Shanxi. Compared with haplotypes of susceptible strains (hap5 and hap9‐11, hap13 in Figure 3; Van Leeuwen et al., 2008), no novel amino acid substitution was found, supporting the notion that TSSM with hap2 is susceptible to bifenazate. Three nonsynonymous substitutions were identified in the other three haplotypes of *cytb*. Compared with hap2, G126S/G376A (protein/gene) substitution occurred in Hap3, which is located at the Qo site of *cytb*, and cd1 helix in cytochrome bc1 complex. This mutation has been related to resistance of TSSM to bifenazate, causing moderate levels of resistance (Van Leeuwen et al., 2008). G126S is mainly distributed in populations with medium resistance levels, with a frequency of 100% in Beijing and 72% in Sichuan populations. A novel mutation, A269V/C806T, was found in Hap1, which is present in the population from Anhui (50% in frequency) and Hunan (15% in frequency) with low‐level resistance The mutation I86M/T258A occurred in hap4 and is mainly distributed Zhejiang with a frequency of 47%. It has been reported in a laboratory‐selected resistant strain to bifenazate (HOL2; Hap7 in Figure 3; Van Leeuwen et al., 2008). This mutation did not increase levels of resistance caused by G126S and, thus, is unlikely to be related to bifenazate resistance (Van Leeuwen et al., 2008).

### 3.3 Population genetic structure

We obtain 155 unique genotypes among the 170 female individuals of TSSM characterized for the five microsatellite loci. The average allelic richness (*A*_R_) varied from 2.424 to 3.159. The observed heterozygosity (*H*_o_) is lower than expected heterozygosity (*H*_e_) in all populations except for one case (Table 4). The inbreeding coefficients (*F*_IS_) are positive except for one population (Table 4) suggesting mattings between relatives. Pairwise *F*_ST_ values among populations ranged from 0 to 0.2885 (Table 5). Four out of 50 population‐locus pairs show deviation of Hardy–Weinberg equilibrium (HWE; *p* < 0.05); however, none locus show deviation in all population or population show deviation on all loci (Table 6).

**Table 4:** Genetic diversity parameters in populations of *Tetranychus urticae* based on microsatellite loci

Population	*A*_R_	*A*_T_	*H*_e_	*H*_o_	*F*_IS_
SCCD	2.9812	18	0.5774	0.4170	0.1893
HNCS	2.4978	13	0.4810	0.3817	0.2627
AHHN	2.5196	13	0.4834	0.3589	0.1046
ZJJX	3.0692	16	0.5883	0.3312	−0.0129
SXYQ	3.1590	17	0.5314	0.4201	0.1950
SDRZ	2.8332	15	0.5098	0.4012	0.2437
BJC1	2.6582	17	0.4698	0.3830	0.2817
BJC2	2.4242	14	0.4423	0.3989	0.2028
BJCW	2.7854	17	0.4996	0.5048	0.2154
BJDX	2.6330	15	0.4972	0.4036	0.4472

Note

For population codes see Table 1.

*A*_R_: average allelic richness;* A*_T_: total number of alleles; *F*_IS_, inbreeding coefficient;* H*_e_: expected heterozygosity; *H*_o_: observed heterozygosity.

**Table 5:** Pairwise *F*_ST_ among *Tetranychus urticae* populations based on microsatellite loci

Population	SCCD	HNCS	AHHN	ZJJX	SXYQ	SDRZ	BJC1	BJC2	BJCW
HNCS	0.0575*								
AHHN	0.0046	0.1373**							
ZJJX	0.0605**	0.0908**	0.1388**						
SXYQ	0.1534**	0.2885**	0.2068**	0.0693**					
SDRZ	0.1495**	0.1908**	0.2042**	0.1922**	0.2467**				
BJC1	0.0855**	0.1058**	0.1026**	0.1558**	0.2565**	0.0978**			
BJC2	0.1002**	0.1571**	0.1140**	0.1384**	0.2166**	0.1034**	0.0000		
BJCW	0.0652**	0.1394**	0.0705**	0.1043**	0.1801**	0.1203**	0.0041	0.0000	
BJDX	0.0640**	0.0881**	0.0933**	0.0955**	0.2102**	0.0707**	0.0000	0.0000	0.0000

For population codes see Table 1.

* *p* < 0.05.

** *p* < 0.01 following Holm's correction.

**Table 6:** *p*‐Value of Hardy–Weinberg equilibrium in populations of *Tetranychus urticae* of five microsatellite loci

Population	S05	S158	S167	S19	S65
SCCD	0.0760	0.0269	0.7659	0.0598	0.1662
HNCS	0.0607	0.4414	0.1796	0.4940	1.0000
AHHN	0.0008	0.3148	0.0799	0.6114	1.0000
ZJJX	0.3585	0.3097	0.0093	0.1322	0.0019
SXYQ	0.0170	0.0000	0.7853	0.0408	0.0291
SDRZ	0.5807	1.0000	0.0475	0.4402	1.0000
BJC1	0.4078	0.7168	0.3759	0.1620	1.0000
BJC2	0.7551	1.0000	0.4574	0.2715	1.0000
BJCW	0.0185	0.6441	0.3870	0.3860	0.7210
BJDX	1.0000	0.2393	0.1192	1.0000	0.3193

Note

S05, S19, S65, S158, and S167 represent five microsatellite loci, respectively, which are referred in previous work about microsatellite development in TSSM (Ge et al., 2013).

**Figure 4:**
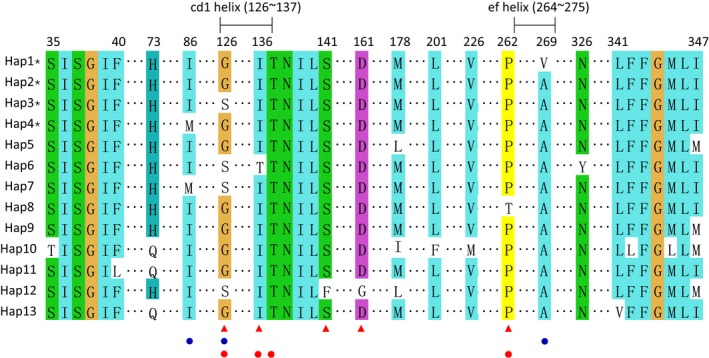
Phylogenetic relationships (a) and discriminant analysis of principal components of population genetic structure of *Tetranychus urticae *based on microsatellite loci for all populations (b) and populations when outlier populations of SXYQ and ZJJX were excluded (c). Points with the same colors are individuals from the same population

Phylogenetic analysis showed that five northern populations grouped into a major lineage, while the southern populations grouped into another major group (Figure 4a). BAPS analysis based on microsatellite loci divided all populations into three clusters (Figure 4b). One cluster mainly included five northern populations. Another cluster was mainly distributed in the Shanxi population and partially in the Zhejiang population. The other cluster was widely distributed in southern populations. Zhejiang population was composed of all three clusters.

Discriminant analysis of principal components analysis on all populations indicated two populations from Shanxi and Zhejiang as outliers (Figure 4b). When we excluded these two outlier populations, four northern populations from Beijing clustered into one group, leaving one population from Shandong and three southern populations around them, although the relationships of these populations are not very clear (Figure 4c). The pattern of population genetic structure identified from DAPC is similar to that inferred by BAPS analysis.

### 3.4 Correlation between genetic differentiation and geographical distance and resistance level

Mantel tests showed there was no correlation between genetic distance and geographic distance (*r* = 0.13, *p* = 0.25) or between these measures and resistance to bifenazate (*r* = −0.149, *p* = 0.759). No correlation was found between resistance to bifenazate and the membership coefficient matrices (*Q*‐matrices) when the optimal K was 2 (P1 = 0.089, P2 = 0.4).

### 3.5 Gene flow

**Figure 5:**
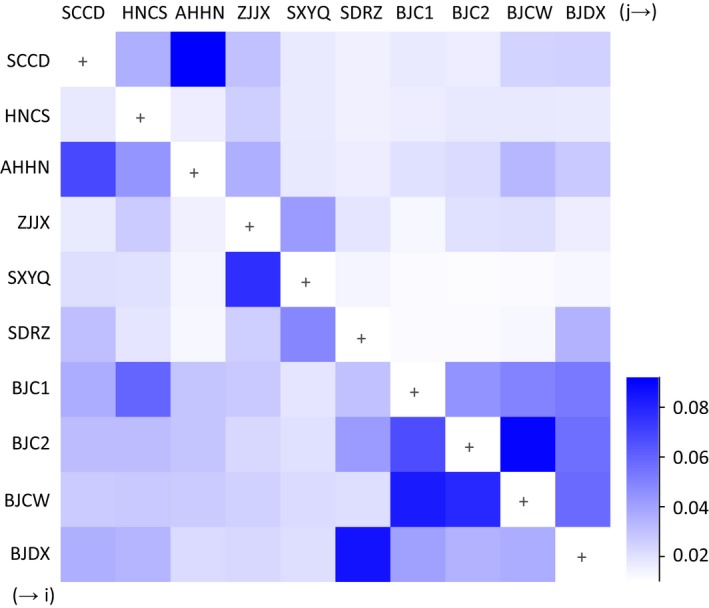
Heatmap of gene flow among ten field populations of *Tetranychus urticae* across China based on microsatellites estimated using BAYESASS. Dark color indicates high levels of gene flow from population (j)–(i), while the light color indicates a low level of gene flow

Relatively high levels of gene flow were found among populations from Beijing, especially among three populations from Changping of Beijing (BJCA, BJCB, and BJCW with mean value of m ranged from 0.067 to 0.09). High level of gene flow was also found from AHHN to SCCD (*m* = 0.092, 95% HPD = [3.750E‐6, 0.186]), from SCCD to AHHN (*m* = 0.069, 95% HPD = [2.972E‐7, 0.153]), from ZJJX to SXYQ (*m* = 0.077, 95% HPD = [8.851E‐7, 0.138]), and from SDRZ to BJDX (*m* = 0.086, 95% HPD = [1.137E‐7, 0.171]; Figure 5).

## 4 DISCUSSION

### 4.1 Bifenazate resistance and mutation of *cytb* gene

Compared with the laboratory‐selected resistant strain (BR‐VL strain, resistance ratio >164,000; Van Leeuwen et al., 2008), resistance is not extremely high in TSSM populations from China. However, resistance has developed rapidly, especially in the Beijing and Sichuan regions where there are major areas of strawberry production and nursery activities that need frequent control of TSSM. Resistance detected in our study is congruent with the poor control of TSSM by bifenazate in these regions.

We found four haplotypes of the target *cytb* gene with three mutations compared to the susceptible haplotype (Van Leeuwen et al., 2006, 2008). Mutation G126S was found in five populations that had about 20‐fold to 30‐fold resistance, consistent with previous findings indicating that this mutation leads to medium resistance to bifenazate in TSSM (Van Leeuwen et al., 2008). G126S is currently the major mutation affecting bifenazate resistance in China. Mutation I86M was found exclusively in Zhejiang population, where it was at an intermediate frequency but did not seem to increase the resistance level of this population compared with two populations with the susceptible haplotype, consistent with previous work that this mutation does not result in resistance in TSSM (Van Leeuwen et al., 2008). Declined susceptibility in populations with low LC50 values (twofold to fourfold over the susceptible strain) may be caused by metabolic mechanisms or error of bioassay. The resistance ratio can be influenced by the susceptibility strain used for comparison. The susceptible population was reared in laboratory for many rears without any contact with pesticide. The laboratory adaptation may in the population and lead to increase in susceptibility to pesticides (Hoffmann & Ross, 2018). A novel mutation of A269V was also found in the present study; it is located in the ef helix aligning in cytochrome bc1 complex, though not at the conserved site (Esser et al., 2004). Populations from Anhui and Hunan with the A269V mutation tend to have some resistance to bifenazate, compared to both susceptible strain and three field populations; and the more frequent the A269V mutation, the higher the resistance level. We therefore suspect that the A269V mutation may be associated with a low to medium level of resistance to bifenazate. However, metabolic mechanism may also lead to the resistance of TSSM to bifenazate, which need further validation.

### 4.2 Standing genetic variation in resistance mutation

The major mutation G126S that confers resistance of TSSM to bifenazate in China was previously detected in the Beijing area prior to the application of bifenazate in 2013 (Gong et al., 2014). The frequency of this mutation has increased from 1/288 to a very high frequency (100% in four Beijing populations) within five years. The G126S mutation showed obvious selective advantage in resistance populations, which support that the G126S is a resistant mutation in the TSSM to bifenazate as previously reported (Van Leeuwen et al., 2008). The G126S mutation is common in TSSM populations worldwide (Van Leeuwen et al., 2008) as well as being present in multiple populations of citrus red mite *P. citri* (Van Leeuwen et al., 2011). This suggests standing genetic variation for bifenazate resistance in mite populations. Combination of G126S with other mutation appears to have limited fitness costs (Van Leeuwen et al., 2008), and this likely contributed to the presence of G126S in natural populations prior to selection through bifenazate. Testing for this mutation in a production area prior to the application of bifenazate should help in assessing the risk of resistance developing when implementing an IRM program.

### 4.3 Multiple origins of resistance mutations

A phylogenetic approach has been used to identify the origin of resistance alleles in this species (Hawkins et al., 2018). Here, we used a population genetic analysis to examine the population structure of TSSM and infer the origin of resistance mutations similar to the method of using a haplotype network (Karasov, Messer, & Petrov, 2010). Overall, we found that nearby populations of TSSM are likely to belong to the same genetic cluster, although the IBD analysis of pairwise genetic differentiation (*F*_ST_) and geographic distance of TSSM populations showed no significant correlation when the entire data set was considered. Here, we used five microsatellite loci for population genetic analysis due to rare microsatellite loci in the small genome of TSSM (Grbic et al., 2011; Saune et al., 2015) and limited template DNA from each individual. This may bias the estimation of gene flow. However, five microsatellite loci were able to reveal the population genetic structure of a species as in previous study (Meng, Shi, & Chen, 2008). The genetic structure described here is consistent with a previous report that indicates spider mite populations represent metapopulations (Osakabe et al., 2009).

Two major clusters are located in north and south China, with relatively strong genetic differentiation (*F*_ST_) and low gene flow between them. This is consistent with a previous study which found high genetic differentiation and strong evidence for limited gene flow among geographically separated populations (Sun et al., 2012; Xie et al., 2006) though different locations were sampled. The resistance mutation G126S was detected in two genetic clusters from southern and northern China. This may indicate the independent origin of G126S in these two groups of distantly related populations. However, the Sichuan population overlapped with the major cluster in the northern area, suggesting gene flow and the potential movement of resistance mutations. It is also clear that two southern populations had different resistance alleles and fell into genetic clusters separate from the northern populations, whereas the five northern populations fell into the same genetic cluster but had different mutations. These results point to the independent origin of resistance mutations in TSSM populations. The independent origin of resistance may involve various factors such as the initial frequency of resistance genes, intensity of selection through pesticide applications, dispersal patterns, and habitat stability (Osakabe et al., 2009).

### 4.4 Accelerating the rate of resistance development by gene flow

Resistance to bifenazate developed rapidly in populations from Beijing that attained a medium level of resistance from 2013 to 2017, more rapidly than in other regions. Local dispersal leading to high gene flow may account for this pattern (Saavedra‐Rodriguez et al., 2015), particularly as four populations from Beijing share the same resistance mutation and come from the same genetic cluster. The pairwise *F*_ST_ values are very low while gene flow estimates are high, ranging from 0.035 to 0.090. Regional dispersal of spider mites can occur from wind‐assisted movement and passive transport from human movement including agricultural practices (Osakabe et al., 2009; Uesugi, Kunimoto, & Osakabe, 2009). Importantly, strawberry seedling transportation is an essential way of TSSM dispersal and is likely to spread resistance mutations. The location of the BJCW population covers the main production nursery of strawberries, and seedlings are transported to other planting areas around Beijing, promoting resistance dispersal.

Dispersal linked to cross‐regional transportation also likely accounts for genetic similarity between Beijing and Sichuan populations. From the perspective of mitochondrial genes, the Beijing populations are all classified as haplotype hap2, the same haplotype to which most individuals of the SCCD population belong to, while resistance level of both Beijing and SCCD populations is consistently high. From the perspective of microsatellite data, there is also relatedness between SCCD individuals falling into cluster 2 and the Beijing populations. Gene flow from SCCD to Beijing was estimated as 0.033 on average, while reciprocal gene flow had an average estimate of 0.021. This gene flow level seems likely to reflect cross‐regional transportation between Beijing and Sichuan populations; Sichuan Province is a major production area for strawberries, and seedlings as well as other products are transported to different regions in China. Beijing is an international metropolis and depends on importation of agricultural products from other regions as well as local products. Previous reports have noted long‐distance dispersal by TSSM consistent with this notion (Osakabe et al., 2008).

Nevertheless, a high level of gene flow does not necessarily mean an equivalent level of resistance. In particular, gene flow between Sichuan and Anhui was estimated as extremely high (from Sichuan to Anhui the average estimate was 0.069, while in the reciprocal direction it was estimated as 0.092). Yet, resistance to bifenazate was at a different level in these two locations. This may reflect differences in selection pressures in these regions, but this remains to be tested with additional sampling.

## 5 CONCLUSION

Resistance of TSSMs to the novel acaricide bifenazate is developing in China. Taking advantage of the well‐studied genetic basis of bifenazate resistance, we monitored bifenazate resistance among TSSM populations and compared results to the population genetic structure of TSSM populations. The major resistance mutation in TSSM was probably present in populations before pesticide‐related selection, although it appears to have evolved independently in populations of TSSM in China. Nevertheless, within a region and likely also across regions, gene flow among populations appears to have accelerated the development of resistance. Our study therefore suggests that the origin and development of pesticide resistance in fields can depend on local selection pressures as well as on movement. These results are relevant to IRM strategies in remaining regions where resistance has not yet developed. Populations from these regions should be screened extensively for the major mutation affecting resistance. If present, attempts should be made to reduce local selection pressures. If the mutation is not found, a low level of resistance might still be expected, but it becomes important to reduce gene flow from resistant populations from other regions.

## CONFLICT OF INTEREST

None declared.

## AUTHOR CONTRIBUTIONS

Shu‐Jun Wei conceived and designed the experiments; Ya‐Jun Gong and Jin‐Cui Chen organized the collection of specimens; Pan shi performed the molecular analyses; Pan Shi, Li‐Jun Cao, Wei Song, and Ling Ma analyzed the data; Shu‐Jun Wei, Ary Hoffmann, and Pan Shi discussed the results; Shu‐Jun Wei, Pan Shi, and Ary Hoffmann wrote the paper.

## Data Availability

Data available from the Dryad Digital Repository: https://doi.org/10.5061/dryad.qb701kq.
